# Workplace changes, perceived difficulties and migration intentions among Romanian construction workers during the covid-19 pandemic

**DOI:** 10.1371/journal.pone.0335155

**Published:** 2026-06-24

**Authors:** Vasile Chasciar, Denisa Ramona Chasciar, Claudiu Coman, Vasile Gherhes, Cristel Iotu, Adrian Netedu

**Affiliations:** 1 Faculty of Social Sciences, University of Craiova, Craiova, Romania; 2 Faculty of Psychology and Educational Sciences, Babeș-Bolyai University of Cluj-Napoca, Cluj-Napoca, Romania; 3 Academy of Romanian Scientists, Faculty of Sociology and Communication, Transilvania University of Brașov, Brașov, Romania; 4 Interdisciplinary Research Center for Communication and Sustainability, Department of Communication and Foreign Languages, Politehnica University of Timișoara, Timișoara, Romania; 5 Faculty of Social Sciences, Doctoral School of Social Sciences and Humanities, University of Craiova, Craiova, Romania; 6 Faculty of Philosophy and Social-Political Sciences, University “Alexandru Ioan Cuza”, Iași, Romania; Università degli Studi di Milano: Universita degli Studi di Milano, ITALY

## Abstract

The COVID-19 pandemic disrupted labour mobility across Europe, but its effects varied substantially across sectors. Construction remained relatively active in Romania, creating a specific context in which workers faced health-related constraints while maintaining employment continuity. This article examines how construction workers in Brașov County perceived workplace changes, pandemic-related difficulties and migration intentions during the COVID-19 pandemic. The study is based on a questionnaire survey conducted among 384 construction workers, with data collected online in February–March 2022. Descriptive statistics were used to summarise workers’ perceptions, working conditions and migration intentions, while chi-square tests of independence and Spearman’s rank-order correlation were applied to examine associations between sociodemographic variables, perceived difficulties, financial impact and willingness to work abroad. The findings indicate that most respondents reported limited changes in workload and working conditions, while the main difficulties were related to mask wearing, compliance with sanitary rules and extended project completion times. Although the overall intention to work abroad was low, perceived financial impact was positively associated with willingness to migrate. The study contributes worker-level evidence on labour mobility intentions in an essential sector during a period of restricted international movement.

## 1 Introduction

Labour migration is a global phenomenon with significant social, economic and political implications, affecting both countries of origin and destination. Its main drivers include economic inequalities, limited employment opportunities, low wages, precarious working conditions and lack of professional prospects, particularly for workers with medium or low levels of education [1,2].

The exodus of the workforce has multiple consequences. On the one hand, countries of origin are facing the loss of human capital, the ageing of the working population and imbalances in the labour market [3]. On the other hand, migrants can contribute to national economies through remittances, transfer of skills and know-how, provided that there are effective policies for professional and social reintegration [4]. At the same time, destination countries benefit from filling labour shortages, especially in less attractive sectors, although integration challenges such as discrimination, job insecurity and cultural adaptation persist [5].

A further structural effect is the “brain drain” phenomenon, particularly pronounced in Eastern European countries, where the emigration of skilled workers affects key sectors such as health, education and infrastructure [3].

The construction sector is among the most affected by labour mobility. Characterised by seasonality, demanding working conditions and a constant need for labour with varying qualifications, construction has become highly dependent on migrant workers. In the European context, countries such as Germany, Great Britain and France have been major destinations for workers from Central and Eastern Europe, particularly Romania and Poland [6]. According to the International Labour Organization, approximately 7% of migrant workers globally are employed in construction, making it one of the sectors most reliant on international labour mobility and simultaneously one of the most exposed to exploitation, informal work and insufficient social protection [7].

In Romania, migration in construction is driven not only by wage differentials, but also by instability in domestic labour supply. Workers often seek better working conditions, higher wages and improved safety standards, which are perceived as more strictly regulated in Western European countries [8]. As a result, labour shortages in construction have become a structural problem, prompting authorities to facilitate the import of non-EU workers to cover domestic deficits.

Labour migration in construction generates significant economic effects at both macroeconomic and microeconomic levels. In the short term, remittances contribute to domestic consumption and poverty reduction. In Romania, remittances have represented between 2–3% of GDP in recent years, with more than six billion euros sent annually, mainly by workers employed in construction, agriculture and services [9]. In the long term, however, sustained emigration may lead to personnel shortages, project delays and declining quality of work execution, particularly when shortages are compensated by insufficiently trained labour [10]. Demographic effects further intensify these challenges, especially in rural areas, placing additional pressure on pension and health systems [3].

For destination countries, the import of labour responds to persistent workforce shortages in construction, a sector marked by seasonal fluctuations and constant demand. At EU level, construction is classified among the areas with a high risk of personnel shortages, and attracting foreign workers is considered essential for maintaining competitiveness and infrastructure development [11]. While migrant labour contributes to GDP growth and real estate expansion, it also raises concerns regarding social dumping and unfair competition, as foreign workers often accept lower wages and harsher conditions [7,12]. Integration difficulties related to language, housing and access to social services remain significant, particularly in contexts with insufficient regulatory control [13].

In Romania, labour mobility in construction has been shaped by legislative measures aimed at facilitating the recruitment of foreign workers, particularly from non-EU countries. The legal framework includes Emergency Ordinance no. 194/2002, Law no. 156/2000 and Ordinance no. 25/2014, while the General Inspectorate for Immigration manages work permits. Consequently, the number of non-EU workers admitted to Romania increased from 10,000 in 2018–100,000 in 2023, supported by simplified procedures, accelerated recognition of qualifications obtained outside the EU [14] and fiscal incentives for employers [15]. From a legal perspective, labour mobility in the construction sector is shaped by a combination of national regulations and European directives, which directly influence recruitment practices, access to the labour market and employers’ reliance on foreign workers.

Similar dynamics can be observed across the European Union. Germany promotes labour immigration through the Skilled Labour Immigration Act [16], while Italy regulates inflows via its annual “Flow Decree” [17]. At EU level, Directive 2011/98/EU establishes a single permit procedure, while Directive 2009/52/EC and the activities of the European Platform against Undeclared Work aim to prevent exploitation and strengthen labour inspections [18,19]. Nevertheless, insufficient control mechanisms continue to generate risks related to undeclared work, labour exploitation and wage inequalities, particularly in construction [20].

The COVID-19 pandemic added further complexity to labour mobility in construction. Global mobility restrictions, quarantine measures and logistical barriers significantly affected both the export and import of labour, temporarily blocking seasonal migration flows and creating operational challenges for recruitment in construction [21]. Although construction was classified as an essential sector in many countries, allowing activity to continue, employers faced delays in supply chains, reduced productivity and persistent labour shortages caused by travel restrictions [22].

**Fig 1:**
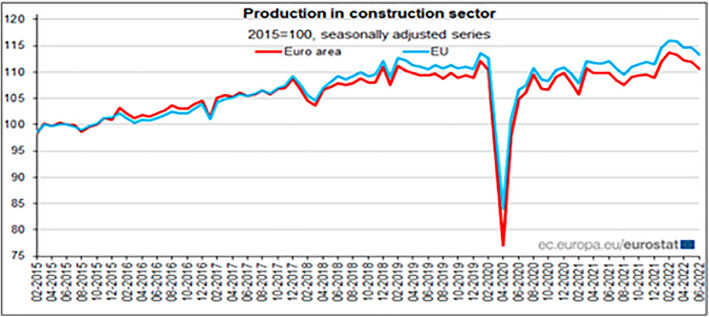
Production trends in the construction sector during the COVID-19 pandemic (Eurostat data).

At the European level, labour mobility in the construction sector followed a distinct pattern compared to other economic fields during the COVID-19 crisis. While many industries experienced significant contraction, construction activity showed a sharp but temporary decline in early 2020, followed by a rapid recovery beginning in 2021. This trend is illustrated in Fig 1, based on Eurostat data, which highlights the resilience of the sector and its essential role in maintaining economic continuity during the pandemic.

These dynamics are relevant for Romania as well, where construction activity continued despite mobility restrictions, creating a paradoxical context: the sector remained operational and in need of labour, while international migration was heavily constrained. Romanian workers encountered additional obstacles such as flight cancellations, quarantine requirements and difficulties in obtaining travel documents [23], while employers increasingly relied on foreign labour, including workers from Southeast Asia [24]. Integration challenges and systemic vulnerabilities in labour mobility were further exposed during the pandemic, emphasising the need for more resilient and inclusive migration policies [25–27].

Although previous analyses have examined labour migration trends and the general effects of the COVID-19 pandemic, there is limited empirical evidence on how Romanian construction workers themselves perceived these changes. Existing studies focus primarily on macro-level indicators, offering little insight into sector-specific experiences at national level.

Building on this gap, the study uses survey data from 384 construction workers in Brașov County to examine workplace changes, perceived difficulties and migration intentions during the COVID-19 pandemic.

Romania is a relevant context for examining labour mobility in construction during the COVID-19 pandemic because labour migration has long been a structural feature of its construction workforce, while the sector also continued to operate during much of the crisis. This created a specific situation in which domestic employment remained relatively stable, but international mobility was constrained by border closures, quarantine rules and administrative uncertainty. Construction workers are analytically important because their work could not be transferred online and because the sector combines project-based employment, physical presence on site, labour shortages and dependence on cross-border mobility. While aggregate indicators such as Eurostat or national statistical data show that construction activity recovered relatively quickly after the initial shock, they cannot capture how workers perceived workplace changes, financial vulnerability, pandemic-related difficulties or the possibility of working abroad. The contribution of this study is therefore to complement aggregate trends with worker-level evidence on temporary immobility and migration intentions in an essential but mobility-dependent sector.

Although labour mobility in construction is shaped by economic, institutional and legal factors, the empirical focus of this study is on workers’ perceptions, workplace experiences and migration intentions during the COVID-19 pandemic. Construction had a specific status during this period, as many activities continued despite restrictions, particularly because work was often performed in open spaces and under conditions that allowed some degree of physical distancing. At the same time, the sector was affected by sanitary rules, mobility restrictions, supply-chain disruptions and changes in work organisation. This article therefore examines how construction workers in Brașov County perceived workplace changes, pandemic-related difficulties and the possibility of working abroad during this period, with particular attention to the relationship between perceived financial impact and willingness to migrate. The study pursued the following objectives:

**O1. To examine the main changes in working conditions and professional activity perceived by construction workers during the COVID-19 pandemic.**
**O2. To analyse workers’ individual perceptions of the COVID-19 pandemic and its effects on their professional life.**
**O3. To assess construction workers’ willingness to work abroad during the pandemic and its association with perceived financial impact.**
**O4. To identify workers’ suggestions regarding measures that could improve working conditions and crisis management in the construction sector.**

Based on the study objectives and the theoretical perspectives discussed above, the empirical analysis tested three associations. These hypotheses are not intended to claim causal effects, but to examine whether workers’ perceptions, difficulties and migration intentions varied systematically according to sociodemographic characteristics and perceived financial impact. This approach is consistent with migration theories that emphasise the role of economic vulnerability, household risk management and structural labour-market conditions in shaping mobility intentions.

- **Hypothesis 1 (H1). Perceived changes in professional life during the COVID-19 pandemic are associated with respondents’ age group and gender.**
- **Hypothesis 2 (H2). The type of difficulty experienced at work during the COVID-19 pandemic is associated with respondents’ level of education.**
- **Hypothesis 3 (H3). Higher perceived financial impact of the pandemic is positively associated with greater willingness to work abroad.**

## 2 Literature review

The COVID-19 pandemic has led to a profound restructuring of labour mobility globally, directly affecting temporary migration regimes and activity in key sectors such as construction. Recent studies reflect both the vulnerability of migrant workers to new health and economic conditions, as well as the need to rethink migration policies in a sustainable manner.

Migration research has traditionally relied on several theoretical approaches that explain why individuals decide to move, how labour markets shape mobility patterns, and why certain sectors, such as construction, become structurally dependent on migrant labour [28,29]. These theories provide the analytical foundation needed to interpret the behaviour and perceptions of Romanian construction workers during the COVID-19 pandemic.

The neoclassical economic theory conceptualises migration as a rational individual decision driven by wage differentials and employment opportunities between countries [30,31]. According to this approach, workers migrate when expected earnings abroad exceed those in the country of origin, after adjusting for migration costs. In Romania, long-standing income gaps between Eastern and Western Europe have historically encouraged the mobility of construction workers toward countries such as Germany, Italy or Spain [32]. This theory helps explain the role of financial considerations in workers’ decisions and is relevant for our hypothesis on the relationship between economic impact and propensity to emigrate.

The New Economics of Labour Migration (NELM) shifts the analytical focus from individuals to households. NELM argues that families engage in migration as a strategy to diversify risk, stabilise income and overcome structural constraints such as unemployment or limited credit access [33,34]. Under this framework, migration decisions are shaped not only by wage differences, but also by household vulnerabilities, many of which were intensified during the pandemic. For construction workers facing income instability, NELM provides a useful lens for understanding how financial pressure may influence the intention to migrate.

Dual labour market theory proposes that labour demand in high-income countries is segmented into primary (stable, well-paid) and secondary (low-paid, unstable, physically demanding) sectors [35]. Migrant workers tend to be absorbed into the secondary segment, which domestic workers avoid due to poor working conditions. Construction is a paradigmatic example of a secondary-sector industry that relies heavily on migrant labour in Western Europe [36]. This theory clarifies why construction was among the first sectors to experience labour shortages during the pandemic and why destination countries continued to rely on migrant workers despite mobility restrictions.

Push–pull models remain widely used to explain the combined influence of factors that push workers away from origin countries (low wages, job insecurity, poor working conditions) and those that pull them toward destination countries (higher salaries, labour shortages, existing migrant communities) [37]. In the Romanian context, limited career opportunities in the domestic construction sector have historically acted as push factors, while strong Romanian diasporas in Germany, Spain and Italy represent powerful pull factors [38]. The pandemic altered both sides of this balance by increasing mobility barriers and health-related risks.

Migration systems theory emphasises the role of social networks, migration histories and institutional linkages in sustaining labour mobility [39]. Established Romanian migrant communities in Western Europe have created transnational systems through which information, expectations and support circulate, shaping migration intentions even during periods of crisis [40].

Taken together, these theoretical perspectives contextualise the empirical findings of the present study. They also support the formulation of our hypotheses concerning the relationship between sociodemographic factors, perceived professional changes, financial impact and migration intentions. By grounding the empirical analysis in established migration theories, the study contributes to a more systematic understanding of labour mobility dynamics in Romania’s construction sector during the COVID-19 pandemic.

Although the literature provides a broad picture of migration in a pandemic context, few studies specifically deal with the impact on the construction sector in Romania. This research gap justifies the need for applied investigations, with a focus on the perceptions of local workers.

A relevant starting point is the analysis carried out by Yeoh, which draws attention to the fragility of temporary migration regimes in crisis conditions [41]. It highlights that migrant workers have often been excluded from the social and economic protection provided during the pandemic, which has raised questions about the long-term sustainability of these schemes. In the same vein, Anderson, Poeschel, and Ruhs propose a rethinking of labor migration, arguing that migrant workers, while essential in maintaining the functioning of certain economic sectors, have been treated as interchangeable labor without adequate recognition of their systemic contribution [42].

International migration has been heavily influenced by movement restrictions and border closures. Podra et al. highlight the decrease in the volume of migration and remittances, especially in Eastern Europe, while Dandekar and Ghai analyze the phenomenon of reverse migration, accentuated in countries with a large diaspora, where workers have temporarily returned to their countries of origin due to job losses [21,43]. These studies indicate that transnational labour mobility cannot be seen only as an economic constant, but as a phenomenon profoundly affected by the global context.

The construction sector has been studied in particular in the literature, being one of the most dependent on the migrated workforce. Pamidimukkala and Kermanshachi explore the impact of the pandemic on workers in this field, showing that both field and administrative staff have experienced significant changes in workload, health regime, and employment uncertainty [44]. At the same time, Stiles, Golightly and Ryan analyze the risks to health and safety in construction, highlighting the additional pressure on employees in the context of insufficient or incoherent protective measures [45].

More generally, the research coordinated by Guadagno and Içduygu highlights the vulnerable situation of migrants during the crisis: from workers stranded in destination countries, to the difficulties of repatriation in the absence of functional bilateral agreements [26,46]. Barker et al. also analyze the impact on the labor market, suggesting that migrant workers were among the first to be affected by the decrease in labor demand, but also by increasingly precarious working conditions [47].

However, most of the studies analyzed offer general or comparative perspectives, without sufficiently capturing the national context or the social dimensions of construction migration. This omission limits the applicability of the conclusions in the Romanian space.

An essential aspect in the analysis of migration in the pandemic is the economic one. Abella and Sasikumar’s estimates of wage losses suffered by migrants bring into question the long-term implications for living standards and the intention to emigrate in the future [48]. In this context, the literature outlines a complex picture of labour migration: a phenomenon that is essential for the balance of the labour market, but profoundly exposed to contextual instability and systemic vulnerabilities.

Overall, recent literature focuses on the interdependence between migration policies, social protection, working conditions and the economic resilience of sectors that depend on migrant labour. The results of our study will be discussed in this theoretical framework to assess the extent to which they align or differ from the conclusions of other research.

## 3 Materials and methods

### 3.1 Sample

The sample consisted of 384 construction workers employed on various construction sites in Brașov County, Romania. The questionnaire was disseminated through site supervisors, who invited workers to complete it voluntarily and anonymously using an online form. This recruitment strategy was chosen because direct access to construction workers was limited during the pandemic and because site supervisors could facilitate contact with workers across multiple construction sites. However, the procedure may have favoured the participation of workers who were more accessible, more willing to respond and able to complete an online questionnaire. This procedure made it possible to reach workers from different construction sites and types of projects, although the sample was non-probabilistic and cannot be considered representative at county or national level.

Brașov County was selected as the research area because it is one of Romania’s dynamic construction regions, combining urban and rural construction sites and showing persistent labour demand in the sector. The local labour market is also connected to national and international mobility flows, which makes it a relevant context for examining workers’ perceptions, pandemic-related difficulties and migration intentions.

The main data collection took place in February and March 2022. Because data were collected in February–March 2022, the responses should be interpreted as late-pandemic and partly retrospective assessments of workers’ experiences rather than as real-time accounts of the initial lockdown period. At the time of data collection, many restrictive measures had already been relaxed compared with the early stages of the pandemic, but workers were still able to report on the professional changes, difficulties and mobility constraints experienced during the broader COVID-19 period. At that time, the target population consisted of 15,097 construction employees in Brașov County, according to official statistical data. The 384 valid questionnaires represented approximately 2.5% of this population. The adequacy of the sample size was checked using G*Power 3.1.9.7. For the statistical tests reported in the study, including chi-square analyses and Spearman correlations, the minimum recommended sample sizes were lower than the achieved sample size, indicating that the sample was sufficient for the planned analyses.

**Fig 2:**
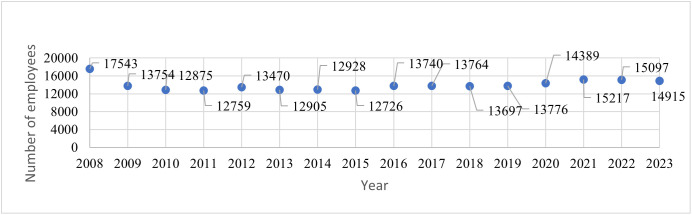
Number of employees in the construction sector in Brașov County, 2008–2023.

The evolution of the number of construction employees in Brașov County between 2008 and 2023 is presented in Fig 2.

It can be observed that the number of construction employees in Brașov County remained relatively stable between 2020 and 2023, with values close to those recorded before and after the pandemic period. This supports the relevance of the county as a context for examining continuity and change in construction employment during the broader COVID-19 period.The main sociodemographic characteristics of the sample are presented in Table 1.

**Table 1:** Main sociodemographic characteristics of the sample.

Variable	Category	n (%)
Age group	Under 35 years	**81 (21.0)**
	35–45 years	**154 (40.0)**
	Over 45 years	**149 (38.8)**

### 3.2 Instrument and data collection

The questionnaire was developed specifically for this study to capture construction workers’ experiences during the COVID-19 pandemic. It included several thematic blocks covering: (a) sociodemographic characteristics (age, gender, education, work experience, area of residence); (b) individual perceptions of the pandemic and perceived changes in professional life; (c) working conditions and specific difficulties encountered during the pandemic; (d) perceived financial impact; (e) intention to migrate for work, reasons for staying in Romania and preferred destination countries; and (f) open-ended questions inviting suggestions for improving working conditions and pandemic-related measures in the construction sector.

Most items were closed-ended questions with predefined response options, including ordinal scales (e.g., questions about the extent to which respondents felt affected financially or the extent to which they would have liked to work abroad during the pandemic), as well as nominal categories summarised in the tables presented in the Results section. The open-ended items allowed respondents to elaborate on their experiences and proposals in their own words, providing contextual qualitative insights that complement the quantitative findings.

The instrument was constructed based on the relevant literature on labour migration, working conditions in the construction sector and the impact of COVID-19 on employment, as well as on the authors’ experience in empirical research in this field. Particular attention was paid to using clear and accessible language, given the diversity of education levels among construction workers. No separate psychometric validation study was conducted, which represents a limitation of the research; however, the questionnaire was designed to align closely with the study objectives and to provide directly interpretable measures for the variables included in the analyses.

Data collection was carried out online using a self-administered questionnaire. Site supervisors distributed the survey link to workers employed on different construction sites, inviting them to participate voluntarily and anonymously. Before completing the questionnaire, all potential respondents received information about the purpose of the study, data confidentiality and their right to refuse or withdraw without any consequences. Only participants who provided informed consent proceeded to answer the questions.

Although the questionnaire was not subjected to a separate psychometric validation study, several steps were taken to ensure its content validity and clarity. First, the structure and wording of the items were developed based on the relevant literature on labour migration, working conditions in construction and pandemic-related disruptions, ensuring alignment with established conceptual dimensions. Second, the initial version of the questionnaire was reviewed by three academic experts in sociology and labour studies, who evaluated the appropriateness, clarity and coherence of the items. Their feedback led to minor adjustments in the formulation of several questions. Third, the instrument was pre-tested informally with a small group of construction workers (n = 12) to assess comprehensibility and the time required for completion; no substantial difficulties were reported, and no further revisions were necessary. While these procedures do not replace full-scale psychometric validation, they provide reasonable support for the internal consistency and applicability of the instrument within the scope of the present study.

The study was approved by the Faculty Council of the Faculty of Sociology and Communication, Transilvania University of Brașov (Decision No. 12 of 3 February 2023). All participants were informed about the purpose of the study, data confidentiality, and their right to withdraw at any time. Informed consent was obtained from all participants prior to participation.

### 3.3 Data analysis

The analysis combined descriptive and inferential statistical techniques. First, frequency distributions and percentages were computed to describe respondents’ perceptions, working conditions and migration intentions. Subsequently, chi-square tests of independence were used to examine associations between categorical variables, such as perceived changes in professional life and age group or gender, and between education level and the main difficulties encountered at work during the pandemic. Effect sizes (phi) were calculated to assess the strength of statistically significant associations.

To test the relationship between perceived financial impact and the desire to work abroad during the pandemic, we used Spearman’s rank-order correlation, given the ordinal nature of the variables. For all inferential analyses, the significance level was set at α = 0.05. The results are presented in Sect 4, accompanied by the relevant contingency tables and correlation coefficients.

## 4 Results

a Individual perceptions of changes generated by the COVID 19 pandemic

From the beginning, the respondents were asked to specify what they associated the pandemic situation/period with expressed as synthetically as possible. The distribution of responses regarding individual perceptions of the pandemic is presented in Table 2.

**Table 2:** Individual perceptions of changes generated by the COVID 19 pandemic.

Perceived change	Frequency	Percent
Affecting health	99	25.8
To be safe	84	21.9
Concerns about the workplace	36	9.4
Restrictions/lack of freedom	33	8.6
To wear a mask	30	7.8
Anxiety/Fear/Panic	18	4.7
Financial problems	15	3.9
I was not threatened	18	4.7
I don’t know/I don’t answer	51	13.3
Total	384	100

We notice that the respondents mostly referred to personal exposure (health problems −25.8%, the fact of being protected −21.9%; anxiety/fear/panic-4.7%) to collective protection measures (restrictions/lack of freedom-8.6%, wearing a mask-7.8%) and less about the professional/work situation (concerns about the workplace-9.4%, financial problems-3.9%). Regarding the fear of health-related risks, only 14% of respondents reported perceiving a possibility of being infected at work (see item Q20 in the questionnaire). These data justify that the construction workers did not have problems with interruption of activity during the pandemic. Moreover, a study published online on the Deloitte website announced that ‘construction defied the pandemic’ and from an economic point of view, the field’s evolution in 2020 was exceptional [33]. The authors showed that large real estate/construction projects continued to develop in Romania despite general economic uncertainties.

In this context, we asked, to what extent has the way you work changed due to the COVID-19 pandemic. This variable was a seven-point Likert scale question with the following descriptive data:

**Table:** 

	N	Minimum	Maximum	Mean	Std. Deviation
Q3. Considering your field of work, to what extent has the way you work changed due to the COVID-19 pandemic?	369	1.00	7.00	2.93	1.36
Valid N	369				

**Fig 3:**
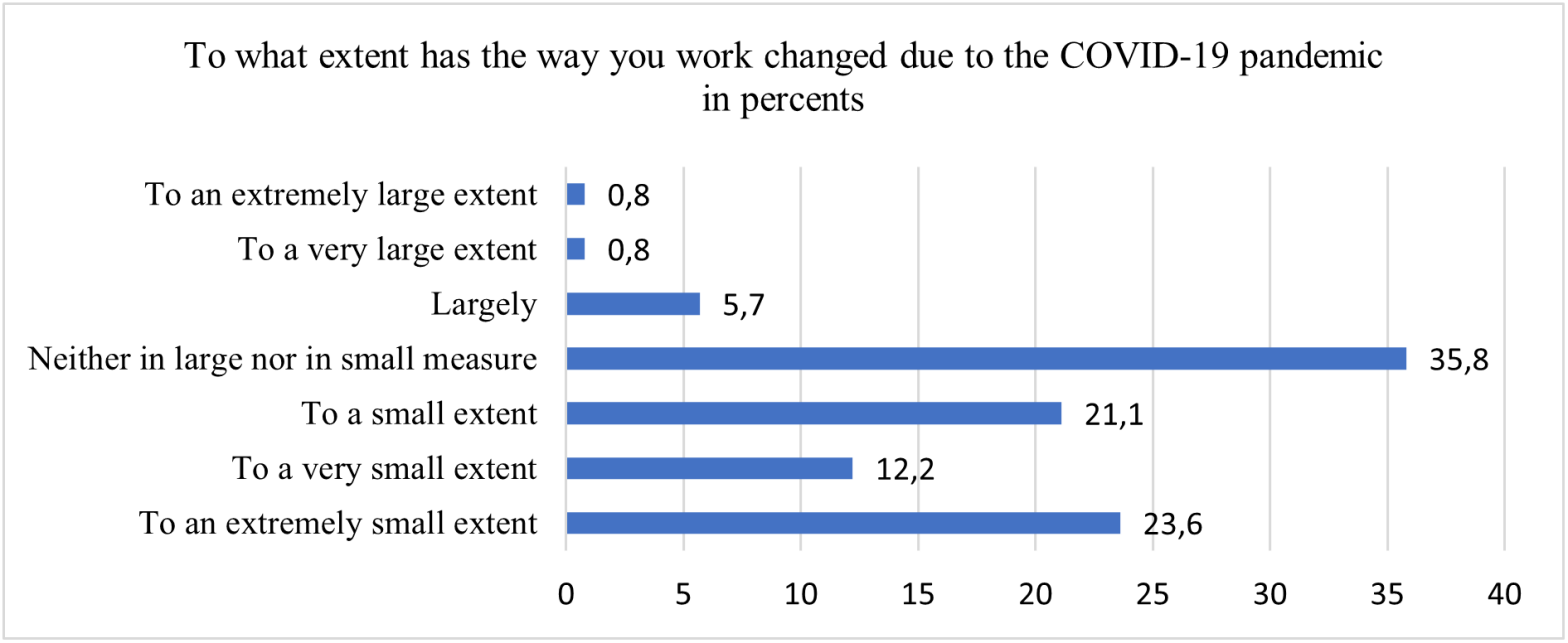
Changes in the way of working caused by the COVID-19 pandemic.

The respondents confirmed that the changes at the place of work were rather reduced in scope (declared a cumulative percentage of 56.9%), as can be seen from Fig 3.

Regarding the amount of work performed during the pandemic, the distribution of responses is shown in Table 3.

**Table 3:** Workload during the COVID-19 pandemic.

Response option	Frequency	Percent
I have worked more than usual	78	20.3
I have worked less than usual	36	9.4
I worked as usual	231	60.2
No answer	39	10.2
Total	384	100

The majority of respondents (60.2%) said they worked at the same pace as before the pandemic, indicating a remarkable level of continuity in their professional activity. In addition, 66.4% reported that the workload during the pandemic was neither harder nor easier compared to the pre-pandemic period. These results suggest that the construction sector has maintained a high degree of stability during the health crisis, probably due to the essential status of the field and the outdoor nature of many construction activities.

To assess satisfaction with remuneration during the pandemic, a seven-point Likert scale was employed. The descriptive statistics for this variable are presented below:

**Table:** 

	N	Minimum	Maximum	Mean	Std. Deviation
18. How satisfied are you with the salary received for the work carried out during the pandemic?	378	1.00	7.00	4.22	1.16
Valid N	378				

**Fig 4:**
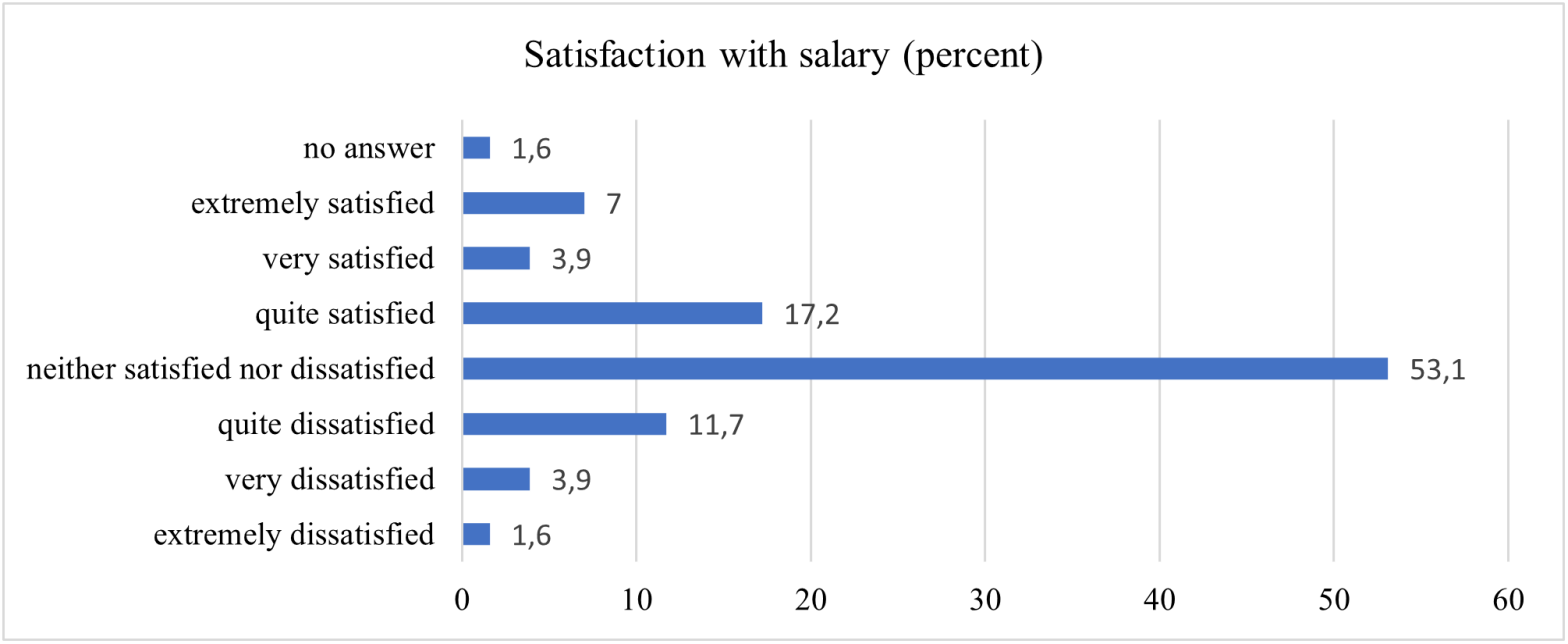
Degree of satisfaction with salary.

In these conditions, the question arises whether the respondents were satisfied with the corresponding salary level. Regarding satisfaction with remuneration during the pandemic, the distribution approaches to a normal distribution, as illustrated in Fig 4.

Satisfaction with pay is predictably balanced with a concentration around average values. From the secondary analyses carried out, we deduced that the remuneration of these employees was growing compared to the pre-pandemic period. Moreover, from the analysis carried out by Ion M. (2022) it emerged that wages and construction production in Romania remained at high levels, the only problem being the acute lack of labour forces. Specialist analyses, said the author, placed Construction in the top of the best-paid fields after IT, Management, Civil/Industrial Design, Research-Development and Project Management.

b **Working conditions and specific difficulties**

The previous results reinforce the conclusions related to the continuity of activity in the field of construction at approximately the same pace. Nevertheless, a series of changes have taken place in the field of construction, as highlighted in Table 4.

**Table 4:** Working conditions and specific difficulties encountered.

Types of changes	Frequencies	Percent
the time for completion of the projects has been extended	135	43.3
the number of workers per shift has decreased	78	25.0
the workload has decreased	57	18.3
the working hours have been shortened	18	5.8
relations with co-workers	66	19.1
relations with superiors	12	3.5
there were no changes	24	7.7

As can be seen, the changes were identified in terms of actual work (completion of the projects, workload, working hours etc.) rather than social relations at work (with colleagues or superiors). The main issue refers to the time for completion of the projects an aspect generally related with the macro structure of the entire economy during the pandemic.

Next, we have separated the working conditions of the respondents according to their declared field of activity, as shown in Table 5.

**Table 5:** Main difficulties experienced at work during the COVID-19 pandemic.

Reported difficulty	Frequency	Percent
Large residential constructions	120	31.3
Small residential constructions	57	14.8
Construction of office spaces	39	10.2
Roads	60	15.6
Civil construction	18	4.7
No answer	90	23.4

In some cases, the dangers of working in construction are higher, a good part of the employees stating that they generally work with large specialized machines (41%) or that they carry out their work at heights (27%).

Added to all this are the inherent difficulties generated by the pandemic situation, which are summarized in Table 6.

**Table 6:** Specific risks in the field of construction and difficulties associated with the pandemic.

Reported difficulty	Frequency	Percent
wearing a mask	225	58.6
carrying out tasks in compliance with the rules for protection against the virus	72	18.8
socializing with colleagues	52	13.5
fulfilling tasks according to the requirements of superiors	23	6
compliance with the work schedule	12	3

c **Propensity to migrate for work**

Considering the inherent difficulties in the pandemic period, we were interested to what extent the respondents were forced to continue working in the country or to try to emigrate (thus accepting the difficulties of moving from one country to another). The reasons for continuing the activity during the pandemic in Romania are presented in Table 7.

**Table 7:** Reasons for continuing to work in Romania during the pandemic.

Reason	Frequency	Percent
I had no other source of income	198	51.6
The risk of illness was lower in Romania	84	21.9
I couldn’t go to another country	42	10.9
Other reason	9	2.3
No answer	51	13.3

The decision to continue working in Romania during the pandemic was mainly driven by financial necessity (51.6%), followed by perceived medical safety (21.9%). Only 10.9% indicated that they would have considered migration if travel had been possible. These responses reveal a combination of structural and subjective motivations: while economic constraints played a dominant role, a significant portion of workers also perceived staying in Romania as a safer personal choice during the global health crisis.

At the European Union level, the construction sector experienced a sharp decline in production at the onset of the pandemic (2020), followed by a remarkable recovery with rising values by 2022. While the industry’s return to near-normal operations could have incentivized international labor migration, mobility remained heavily constrained by stringent pandemic restrictions. To find out that the interviewees from our sample have wanted to migrate even under these conditions, we used a seven-point Likert scale with the following descriptive data:

**Table:** 

	N	Minimum	Maximum	Mean	Std. Deviation
Q17. To what extent would you have liked to have worked in another country in the construction sector during the pandemic?	366	1.00	5.00	2.59	1.44
Valid N	366				

**Fig 5:**
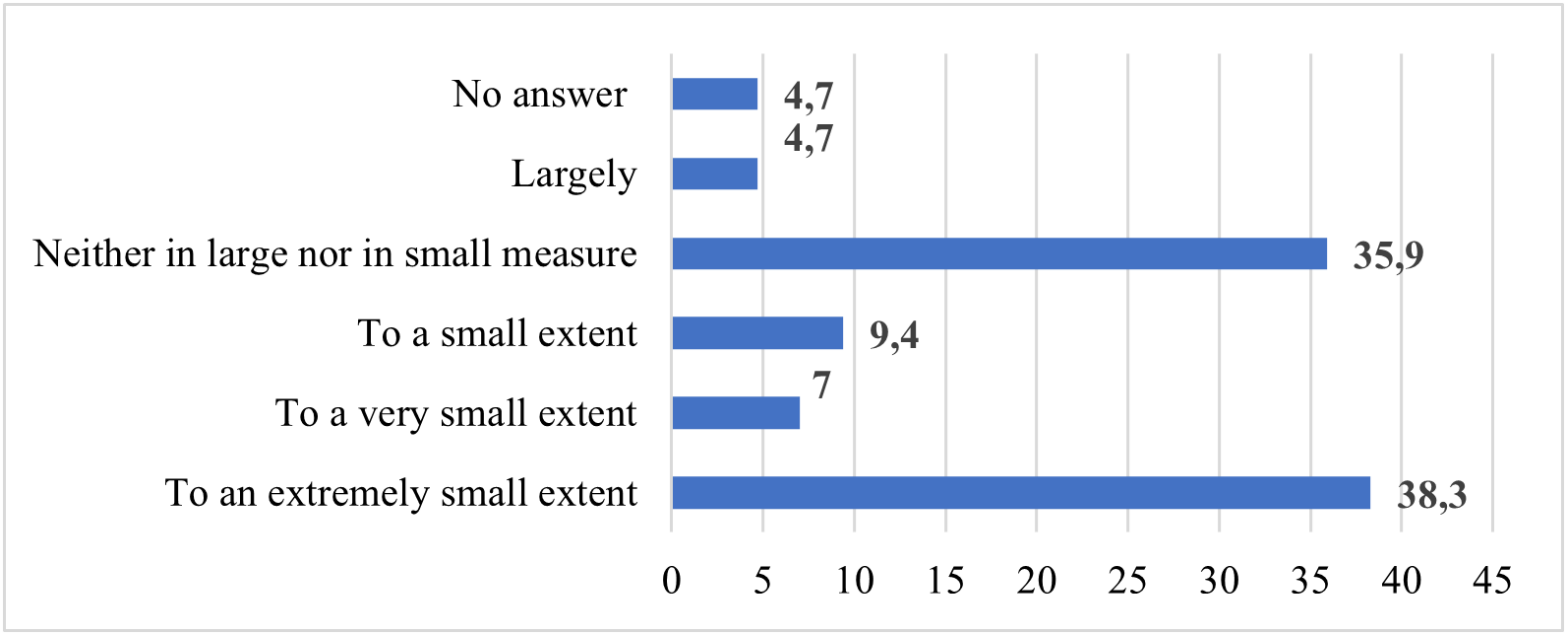
Willingness to migrate for work.

In other words, the construction sector operated at normal parameters during the pandemic, something that could have encouraged international migration, but the restrictions were very firm. Would the interviewees from our sample have wanted to migrate even under these conditions? The distribution of responses regarding willingness to migrate for work is shown in Fig 5.

**Fig 6:**
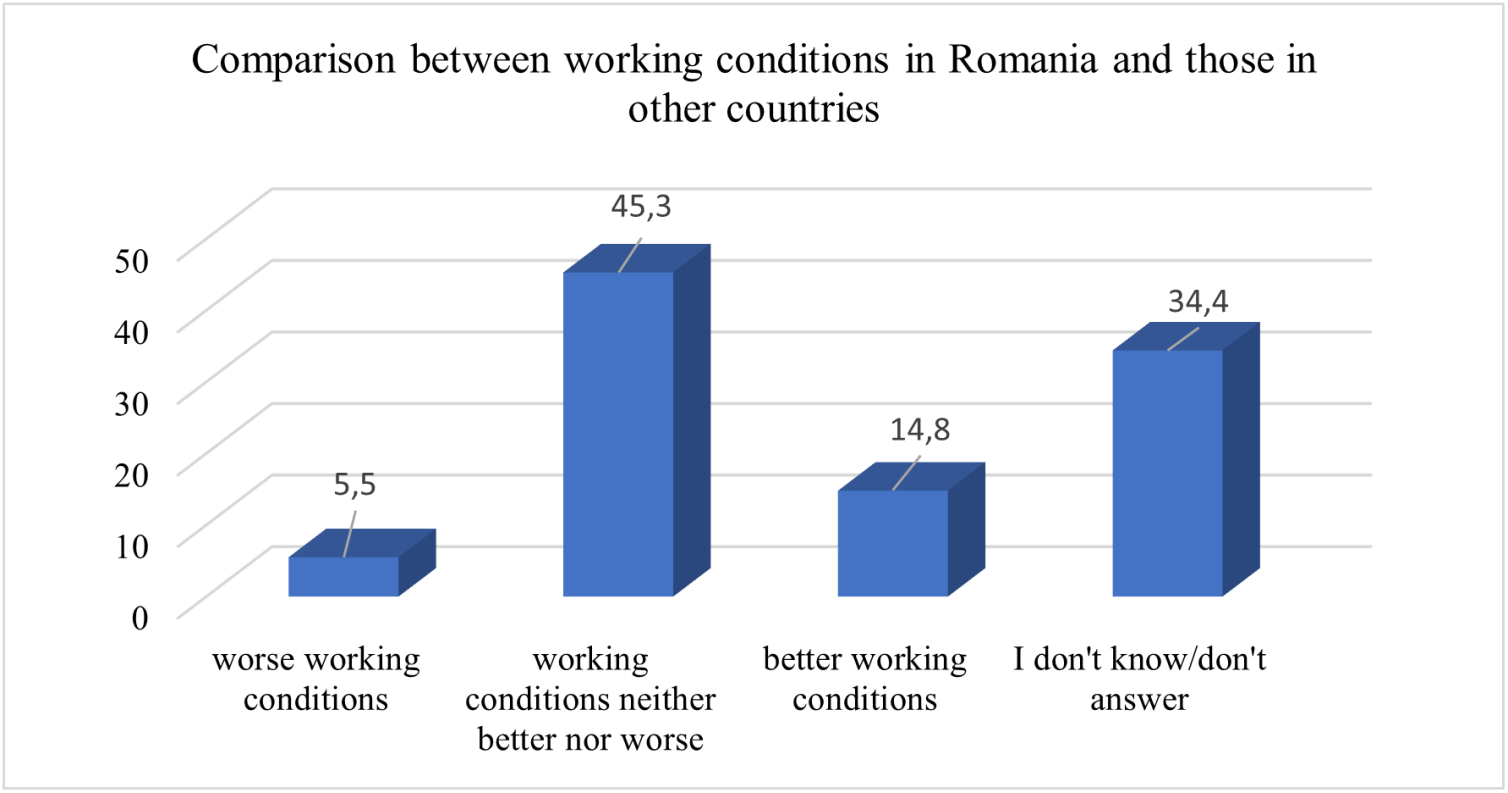
Comparison of working conditions in Romania with those in other countries.

Adding the first three percentages we consider that 54.7% wanted to emigrate to a small and very small extent, 35.9% had a neutral position and only 4.7% wanted to migrate for work. In addition, respondents do not automatically consider that working conditions in other countries are qualitatively superior. The comparison between working conditions in Romania and those in other countries is presented in Fig 6.

As can be seen, 45.3% of the respondents considered the respective conditions comparable, and only 5.5% considered that the conditions in Romania were worse. However, in a hypothetical migration for work, respondents would have preferred, in descending order, Germany (44%), Spain (19%), Italy or the UK (13%), Belgium or Sweden (3%), etc. In all these countries, there are strong Romanian communities of migrants for work.

d **Suggestions and solutions during the pandemic**

Main actors of the construction field, having inside knowledge of the evolution of this field during the pandemic, the sample respondents were invited to state possible solutions for the activity in the field to be improved (for the pandemic period). The first group of suggestions referred directly to the possibility of infection, a fact that reinforces the opinion that even this area was not completely secured (respondents recommended strict compliance with sanitary rules, “avoidance of socialization”, vaccination, isolated work groups with a maximum of 4 employees, sufficient protective materials provided by the state, etc.). One of the respondents summarized some basic requirements “increase in wages, compliance with the rules on the construction site, work schedule with breaks, normal 8-hour schedule, water for workers, better conditions” (male, 47 years old, secondary education). Other interviewees insisted on streamlining interpersonal contacts vertically (with direct superiors) and horizontally (among employees) or recommended staff rotation. The requirement to comply with a normal work schedule is also reflected when some respondents ask for a limit of 10 hours of work (a fact that denotes not only exceeding the conventional eight hours but also faulty time planning).

When asked who should support such pandemic-specific measures, respondents indicated the actors listed in Table 8.

**Table 8:** Respondents’ perception of the responsibility for implementing pandemic-specific measures.

Beneficiary	Frequency	Percent
The State	288	75
Business owners on the real estate market	92	24
Foreign investors	4	1

According to the already mentioned suggestions, the request for the state to be the main decision-maker in these situations (say 75% of the respondents) refers, in their opinion, not only to ensuring normal sanitary conditions in a crisis situation but also to the legislative aspects of work in the field of construction, to the specific controls started by the state authorities, to the price policies and fiscal facilities in the field, to legislative and administrative measures aimed at shortening the periods for issuing authorizations of constructions, to the facility to attract new employees in the field.

### 4.1 Testing hypothesis

**Hypothesis 1. Perceived changes in professional life during the COVID-19 pandemic are associated with respondents’ age group and gender.**

To test this hypothesis, we applied the Chi-Square test of independence and in this case, we used G*Power software to calculate the minimum sample volume that can be used in the analysis. For effect size ω = 0.3, alpha = 0.05, and df = 10, we obtained a minimum sample size of 271 subjects. This sample represents the minimum number of observations to be able to assume the statistical effect of type I error probability and power. We consider satisfactory this value for the minimum volume of the sample (our sample has 384 subjects questioned!). After that, we made a statistical test of independence between the multiple response variable (about main changes found in professional life) and categorical variable of age (recoded with three categories: under 35 years (21% from total sample), between 35 and 45 years (40% from total sample) and over 45 years (the remaining 39% of the sample)). The distribution of responses by age category and type of change in professional life is shown in Table 9.

**Table 9:** Association between the main changes in working life and the age of the respondents.

	Age categories		
	Under	Between	Over
Changes in professional life:	35 years	35 and 45 years	45 years
	Row N %	Row N %	Row N %
workload	16.7%	33.3%	50%
work schedule	39.1%	47.8%	13%
working conditions	26.2%	40.5%	33.3%
relations with superiors	25%	50%	25%
relations with co-workers	18.2%	36.4%	45.5%

We obtained that the two variables were associated (for Pearson Chi Square = 51.328, df = 10, p = .000). We can observe that respondents over 45 years old say that with the pandemic there have been changes mainly in terms of workload (50%). Also, the same people consider to the greatest extent (45.5%) that there have been changes in terms of relations with co-workers. Respondents aged between 35 and 45 years old referred to a greater extent to work schedules (47.8%), working conditions (40.5%), and relations with superiors (50%).

For the case of association between ‘changes in professional life’ and gender variable with G*Power software (for effect size ω = 0.3, alpha = 0.05, and df = 5) we obtained a minimum sample size of 220 subjects. The association between gender and perceived changes in professional life is presented in Table 10.

**Table 10:** Association between changes in work life and gender of respondents.

		Gender	
		male	female
		Row N %	Row N %
Changes in professional life	workload	70.8%	29.2%
	work schedule	87.0%	13.0%
	working conditions	76.2%	23.8%
	relations with superiors	100.0%	0.0%
	relations with co-workers	72.7%	27.3%

The two variables were associated (Pearson Chi Square=12.1, df=4, p=.032). We can observe that the male respondents were dominant in ‘perceptions of changes in professional life’ in all five cases. Finally, we conclude that hypothesis H1 is confirmed: respondents’ opinions regarding “perceptions of changes in professional life” are associated with the respondents’ age categories and their gender.

**Hypothesis 2. The type of difficulty experienced at work during the COVID-19 pandemic is associated with respondents’ level of education.**

To test this hypothesis, we applied the same the Chi-Square test of independence and we used G*Power software to calculate the minimum sample volume that can be used in the analysis. For effect size ω = 0.3, alpha = 0.05, and df = 9, we obtained a minimum sample size of 263 subjects. We consider this minimum size optimal compared to our sample size (384 subjects). First of all, our analysis of association generated within the crosstabulation matrix 6 cells (30.0%) have expected count less than 5. That’s why we recoded the variable (question 22) eliminating the answer variant related to ‘compliance with the work’ schedule. The relationship between education level and the main difficulties faced during the pandemic at work is summarized in Table 11.

**Table 11:** Association between the recoded variable (question 22) and another categorical variable – results after adjustment for Chi-Square test validity.

Variables	N	What was the main difficulties you faced during the pandemic at work?				χ2	df	p
		carrying out tasks in compliance with the rules for protection against the virus	wearing a mask	socializing with colleagues	fulfilling tasks according to the requirements of superiors			
**Studies**						**27.725**	**9**	**.001**
Secondary education	45	12	24	3	6			
High School	114	18	87	9	0			
Vocational school	117	24	63	18	12			
Higher studies	72	21	39	6	6			
Total	348	75	213	36	24			

Considering the results from previous Table, we note that the association of the two variables was statistically significant (χ^2^ (9) = 27.725, p = .001). Thus, we can conclude that the respondents react differently to specification of the main difficulties faced during the pandemic at work. The magnitude of the effect had the value phi = .282 (p = .001), so a value justifying an association of weak intensity but statistically significant.

We noticed that the majority of those who indicated ‘wearing the mask’ as a major difficulty were the respondents with a high school education level (76.3%), those concerned to the greatest extent with ‘carrying out tasks in compliance with the rules for protection against the virus’ were those with higher education (29.2%). The most concerned about ‘socializing with colleagues’ were the respondents with vocational studies (15.4%) and those most concerned with fulfilling tasks according to the requirements of superiors were those with secondary education (13.3%).The second hypothesis of our research was confirmed.

**Hypothesis 3. Higher perceived financial impact of the pandemic is positively associated with greater willingness to work abroad.**

First of all, we calculated the minimum sample size required for this bivariate correlation. For correlation ro H1 = 0.3, alpha = 0.05, and Power = 0.95, we obtained a minimum sample size of 138 subjects. The Spearman correlation between financial impact and the desire to work abroad is reported in Table 12.

**Table 12:** Spearman Correlation Analysis Result for Bivariate Hypothesis Testing.

			To what extent have you been financially affected by the pandemic?	To what extent would you have liked to have worked in another country in the construction sector during the pandemic?
Spearman’s rho	To what extent have you been financially affected by the pandemic?	Correlation Coefficient	1.000	.397^**^
		Sig. (2-tailed)	.	.000
		N	369	360
	To what extent would you have liked to have worked in another country in the construction sector during the pandemic?	Correlation Coefficient	.397^**^	1.000
		Sig. (2-tailed)	.000	.
		N	360	366

**Correlation is significant at the 0.01 level (2-tailed).

Calculating the Spearman correlation coefficient (rho) for the variables Q14 (To what extent have you been financially affected by the pandemic?) and Q17 (To what extent would you have liked to have worked in another country in the construction sector during the pandemic?)we deduced that the correlation was statistically significant, positive, of the same meaning with medium intensity: rho (369) = 0.397, p = 0.000. In other words, the greater the financial impact of construction workers, the greater the propensity for emigration. The third hypothesis of our research was confirmed.

## 5 Discussion

The findings contribute to the literature by showing how macro-level sectoral resilience was experienced at worker level in a mobility-dependent sector. While aggregate indicators document the relative continuity and recovery of construction activity during the COVID-19 period, they do not explain how workers perceived workplace changes, pandemic-related difficulties or migration opportunities under conditions of restricted mobility. The present study addresses this gap by showing that the continuity of activity in construction was accompanied by low migration intentions, moderate perceived workplace disruption and a positive association between perceived financial impact and willingness to work abroad. In this sense, the results complement macro-level evidence with worker-level insights into adaptive immobility during crisis conditions. The continuity of activity in the construction sector is supported by our data, as 60.2% of respondents reported working as usual, and is consistent with previous research and Eurostat analyses showing a rapid recovery of the sector starting with 2021 [8,11].

A distinctive element of our study lies in the way respondents perceive the impact of the pandemic: only 9.4% associated the pandemic with concerns about the workplace, and only 3.9% cited financial problems, suggesting a relative stability of construction revenues in Romania. This finding correlates with studies by Pamidimukkala and Kermanshachi, which showed that construction activity continued with moderate adaptations, but differs from the observations of Barker et al., which point to a sharper decline in labour demand in other regions [44,47].

Beyond the descriptive patterns observed in the data, the findings point toward a phenomenon often described in the literature as the “immobility paradox”. Despite long-standing structural drivers of labour migration in the construction sector, Romanian construction workers displayed a remarkably low intention to migrate during the COVID-19 pandemic. This apparent contradiction can be explained by the interaction of short-term economic stability, perceived health risks associated with international mobility, and institutional constraints imposed by travel restrictions. While construction activity remained largely uninterrupted and incomes were relatively stable, the uncertainty and logistical barriers surrounding cross-border movement temporarily reduced the attractiveness of migration, leading to a form of adaptive immobility rather than permanent attachment to the domestic labour market.

Regarding the intention to emigrate, our research highlighted a low propensity to migrate during the pandemic: only 4.7% expressed a clear desire to work in another country. This trend is explained by the lack of alternatives (51.6%) and the perception of a higher risk abroad (21.9%). These data align with Guadagno’s and OECD (2022) studies, which observed a decrease in international mobility due to restrictions and uncertainties [4,26]. At the same time, there is a difference compared to research conducted in other contexts, such as India or South Asia, where reverse migration was more strongly accentuated [21,46].

The fact that only 4.7% of respondents expressed a clear intention to work abroad during the pandemic should therefore not be interpreted as an absence of migration aspirations. Rather, it reflects a context-dependent decision shaped by temporary constraints and perceived risks. Financial necessity, the continuation of domestic employment and limited international mobility combined to discourage migration, even in a sector traditionally characterised by high levels of cross-border movement.

A relevant aspect is the relationship between the financial impact and the desire to emigrate, where we found a significant positive correlation (Spearman rho = 0.397, p < 0.01), supporting the hypothesis that economic difficulties increase the intention to migrate. This conclusion is convergent with the data presented by Abella and Sasikumar, who estimated significant wage losses among migrant workers and their correlation with relocation intentions [48].

Regarding the difficulties encountered, “wearing a mask” and “observing sanitary rules” were the main problems mentioned by respondents. This confirms the observations of Stiles et al., who draw attention to the tensions between health protection rules and the often demanding physical nature of construction work [45].

On the other hand, our study also brings into question a form of professional resilience among construction workers, who, despite the pandemic discomfort, have maintained their activity and have not expressed a marked dissatisfaction with their salary or working conditions. This aspect is not sufficiently discussed in the international literature and represents a possible future research direction.

While the results confirm a relatively high level of professional resilience among construction workers during the pandemic, they also point to systemic vulnerabilities that require intervention. Public authorities and employers must work together to ensure the protection and integration of migrant workers, especially in times of crisis. This includes improving housing and health standards, simplifying administrative procedures for legal employment and applying labour inspection mechanisms.

At the policy level, it is essential to develop proactive strategies that prevent over-reliance on external labour without addressing the root causes of internal labour shortages. Investments in vocational training, retention incentives and better working conditions can reduce migration, while integration programmes can support the sustainability of the imported workforce. In this context, resilience should not only be measured by the adaptability of workers, but also by the ability of institutions to respond.

The resilience of the Romanian construction sector identified in our study is also consistent with macro-level assessments carried out during the pandemic. Eurostat [11] and Deloitte Romania [8] reported that construction experienced only temporary disruptions, followed by a rapid recovery, which mirrors our respondents’ perceptions that their workload and working rhythm remained largely unchanged. At the same time, the difficulties mentioned by participants, especially supply delays and extended project timelines, reflect patterns highlighted in international analyses of construction sector disruptions [7,44]. However, unlike findings from Stiles et al. [45], where health and safety concerns shaped workers’ behaviour more profoundly, respondents in our sample associated the pandemic less with occupational risks and more with personal health concerns, suggesting a differentiated local experience.

The reduced propensity for international mobility observed among Romanian construction workers aligns with broader trends documented across Europe during the pandemic. Studies by Guadagno [26], OECD [38] and Içduygu [46] emphasise that mobility restrictions, administrative barriers and uncertainty contributed to a significant decline in temporary and circular migration. Nevertheless, our data reveal a distinctive mechanism: the deterrent effect was rooted not only in restrictive policies but also in respondents’ perceptions that working abroad entailed higher health risks. This nuance differentiates Romanian workers’ behaviour from cases described by Podra et al. [43] or Dandekar and Ghai [21], where reverse migration occurred primarily due to job loss or economic collapse. In contrast, construction employees in Romania maintained stable employment conditions, leading to a more cautious and risk-averse stance toward migration.

The association between financial vulnerability and migration intentions identified in our study corresponds with established migration theories. The positive correlation between financial impact and willingness to migrate reflects assumptions of neoclassical economic theory [30,31] and NELM [33,34], which posit that economic insecurity increases the incentives for labour mobility. Moreover, the relatively stable working conditions and wages reported by respondents may explain the generally low levels of migration intention, consistent with push–pull dynamics [37,38] where reduced push factors limit mobility. The preference for destinations such as Germany, Italy or Spain echoes migration systems theory [39,40], which emphasises the role of established migrant networks and institutional familiarity in shaping migration flows. Thus, while the pandemic temporarily suppressed migration intentions, the underlying structural drivers remained aligned with classical patterns identified in the literature.

The specific difficulties identified by workers, such as mask wearing, compliance with health rules and changes in project timelines, are consistent with international findings on the operational stress faced by construction workers during COVID-19. Pamidimukkala and Kermanshachi [44] similarly observed increased procedural burdens and disruptions to routine site activities. However, the relatively low emphasis placed by Romanian workers on interpersonal conflicts or supervision problems contrasts with observations by Barker et al. [47], who found that management-employee tensions intensified in some contexts. This suggests that in Romania, organisational structures may have adapted more efficiently, or that workers showed higher tolerance for procedural changes given the sector’s stability and continued employment. These findings also reflect the role of the existing legal and institutional framework, which facilitated labour importation during the pandemic and mitigated the effects of international mobility restrictions.

Finally, the increased reliance on imported labour during the pandemic, noted in official reports [18,24,49,50], resonates with our findings regarding the perceived labour shortages and stable domestic employment conditions. While migration inflows continued even amid global restrictions, integration challenges mentioned in the literature [20,25] were also reflected indirectly in respondents’ suggestions for improved working conditions and clearer organisational procedures. These parallels highlight the dual vulnerability of both domestic and foreign workers in a sector that depends structurally on mobility, even in crisis contexts.

## 6 Limitations

This study has several limitations that should be taken into account when interpreting the results. First, the research was conducted in a single county (Brașov), which restricts the generalizability of the results at the national level. Although Brașov is representative in terms of construction activity, there may be regional differences in the dynamics of the workforce.

Second, the sample was based on voluntary participation, and the questionnaire was distributed online with the support of site supervisors. This recruitment strategy may have introduced selection bias. Workers with limited digital access, lower digital literacy, weaker connections to site supervisors or less stable employment arrangements may have been less likely to participate. As a result, the sample may overrepresent workers who were more accessible, more formally integrated into construction sites and more willing to report their experiences. This limitation is particularly relevant in the construction sector, where informal or precarious labour arrangements may coexist with formal employment.

Thirdly, the questionnaire was based entirely on self-reported data, which may be affected by social desirability biases – respondents could have provided answers that they perceived as socially or professionally acceptable, rather than reflecting their true experiences or opinions.

Finally, the research design was cross-sectional and partly retrospective. Data were collected in February–March 2022, after the most restrictive lockdown period in Romania had passed. Therefore, the findings should not be interpreted as capturing workers’ immediate reactions to the first pandemic shock, but rather as late-pandemic assessments of how the crisis affected their work, perceived difficulties and migration intentions. This timing may have introduced recall bias, as respondents were asked to evaluate experiences that had occurred over the broader COVID-19 period. A longitudinal design, with data collected at multiple points before, during and after the pandemic, would have provided a more comprehensive picture of how the crisis influenced mobility, perceptions and sectoral stability over time.

## 7 Future research directions

Future research should extend the analysis beyond Brașov County to determine whether the patterns identified in this study are consistent across other regions of Romania with different labour market dynamics and development levels. A broader comparative approach, including counties with lower construction activity or stronger dependence on migrant labour, would help clarify whether workers’ perceptions during the pandemic follow similar trends nationally. Another important direction is the implementation of longitudinal studies that could capture changes in workers’ attitudes, migration intentions and working conditions over time, offering a more detailed understanding of how crises shape labour mobility in essential sectors. Future investigations could also explore, through qualitative interviews, how organisational practices, management strategies and workplace cultures influenced workers’ ability to adapt during the pandemic. Finally, comparative analyses with other sectors that remained active during COVID-19, such as agriculture or logistics, may provide valuable insights into the specific vulnerabilities and resilience mechanisms of the construction workforce, contributing to a more comprehensive perspective on labour mobility in crisis contexts.

## 8. Conclusions

This study provides worker-level evidence on how a sample of construction workers from Brașov County perceived professional changes, difficulties and migration intentions during the broader COVID-19 period. Unlike other areas deeply affected by the restrictive measures, the construction sector experienced a relative continuity of activity, which is reflected in the data collected from our respondents.

The results show that most of the workers in the sample continued to work in conditions comparable to those of the pre-pandemic period, without significant decreases in income or dramatic changes in working conditions. This finding confirms the literature that has identified construction as an essential sector, more resilient to external shocks [11,44].

Another important result of the research is the reduced propensity towards international migration among workers, despite the difficulties generated by the pandemic. The reasons given – lack of alternatives, health risks, and travel restrictions – provide a coherent explanation for this behavior, which is in line with the observations of other international studies [4,26]. At the same time, our statistical analysis confirmed that the intention to emigrate is positively correlated with the financial impact felt by workers, suggesting that economic motivations remain the main driver of migration, even in times of crisis.

The research makes a valuable contribution by documenting, from an empirical perspective, the experience of Romanian construction workers in a period of global uncertainty. The data presented can support the development of public policies better adapted to the needs of this professional segment – both in terms of domestic working conditions and regulations on the import and export of labour.

At the same time, the results highlight the importance of strengthening protection systems for migrant workers, especially in essential sectors and vulnerable to external shocks. The pandemic has demonstrated, once again, that migration policies must be flexible, but also focused on human rights and the long-term sustainability of the workforce.

Beyond the theoretical and empirical contributions, the research also provides useful information for the main actors involved in labor and construction policy. For employers in the construction sector, the results underline the importance of maintaining stable and predictable working conditions, even in times of crisis, in order to retain the local workforce and avoid over-dependence on external sources. Policymakers are encouraged to develop strategies that combine local workforce development with responsible labour import programmes, ensuring legal clarity and adequate protections. Finally, NGOs and trade unions should continue to advocate for migrant workers’ rights by providing support mechanisms and monitoring tools, especially in situations where rapid changes (such as a pandemic) create structural vulnerabilities.

Future research could expand this study by including other regions of Romania, to test the consistency of findings across different local labor markets. Additionally, comparative analyses with other vulnerable sectors, such as agriculture or HORECA, can provide deeper insight into how different types of essential workforce have responded to the challenges of the COVID-19 pandemic.

In conclusion, our study not only validates a series of trends already reported in the international literature, but also offers a local perspective, relevant for understanding the dynamics of labor migration in Romania and in Central and Eastern Europe in a pandemic and post-pandemic context. These results can inform strategic decisions in the field of human resources, legislative regulations and international cooperation in the field of labour mobility.
